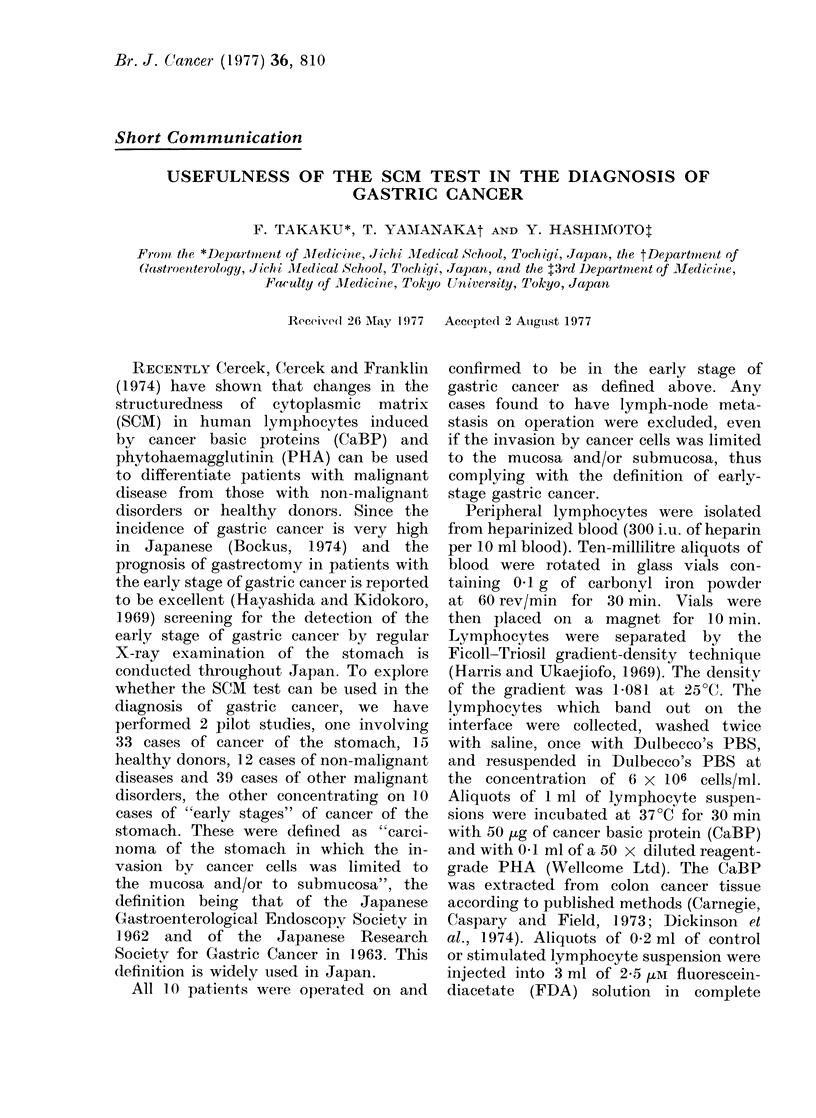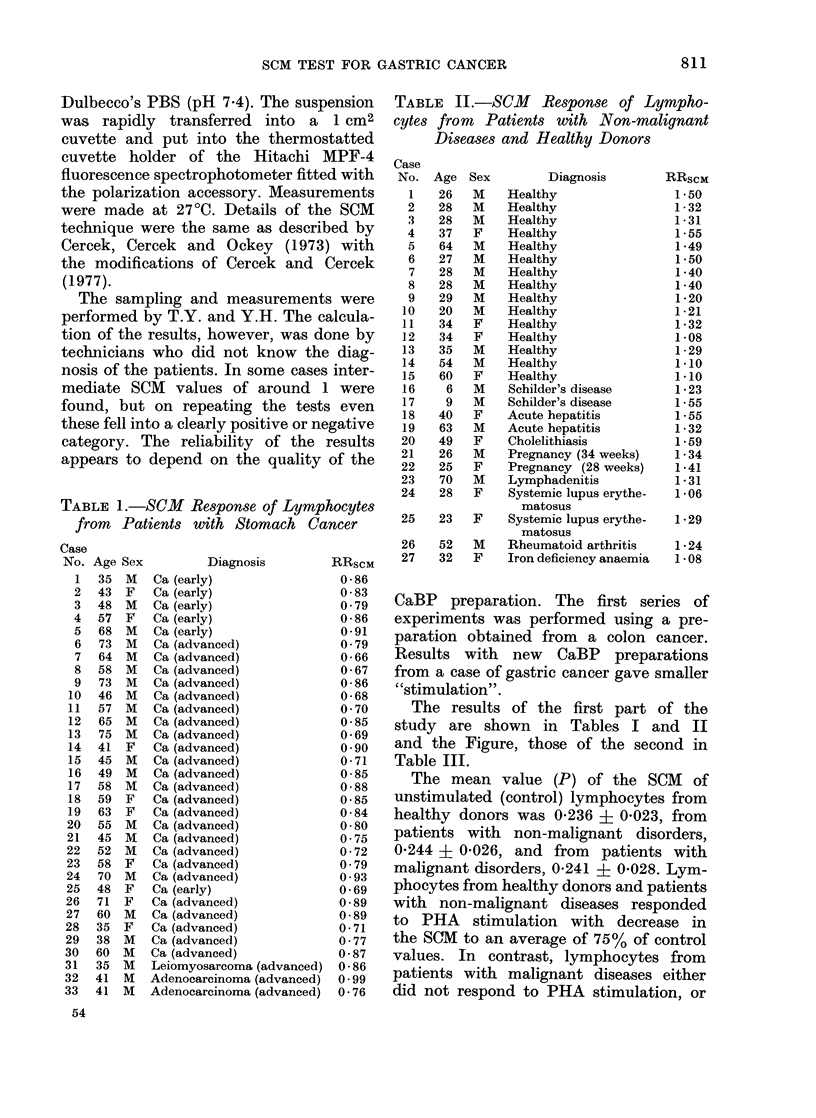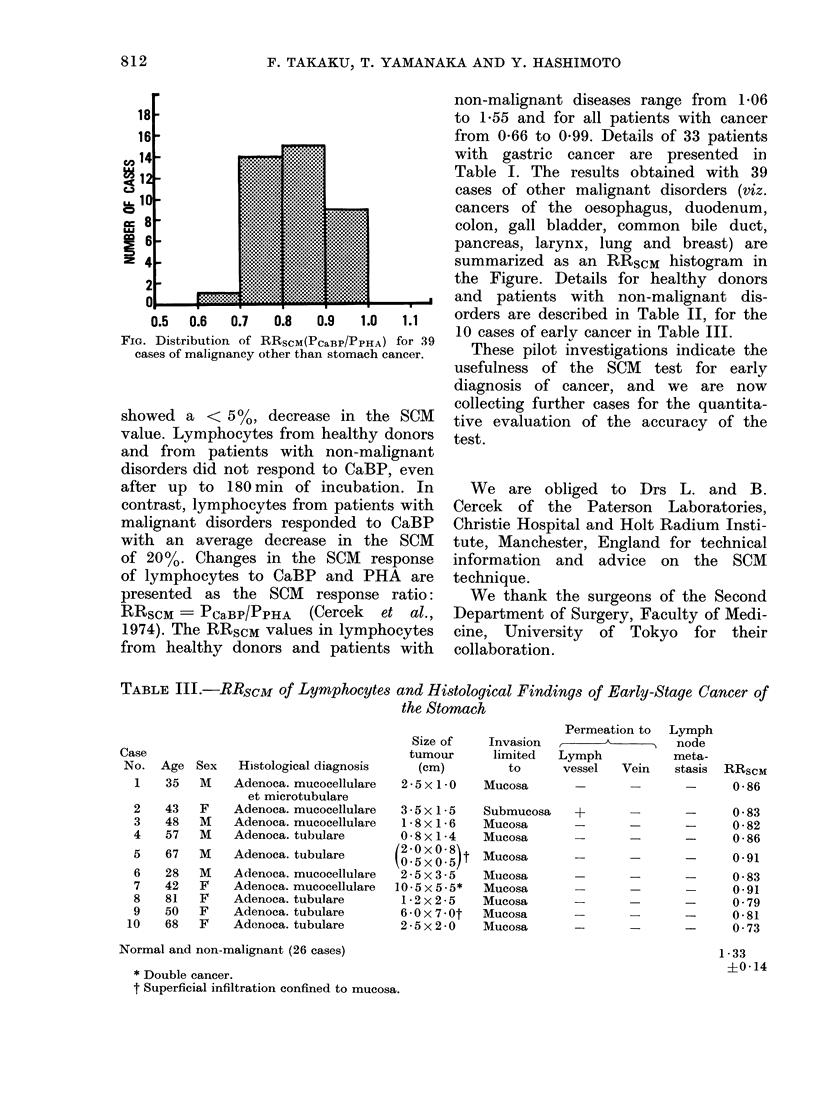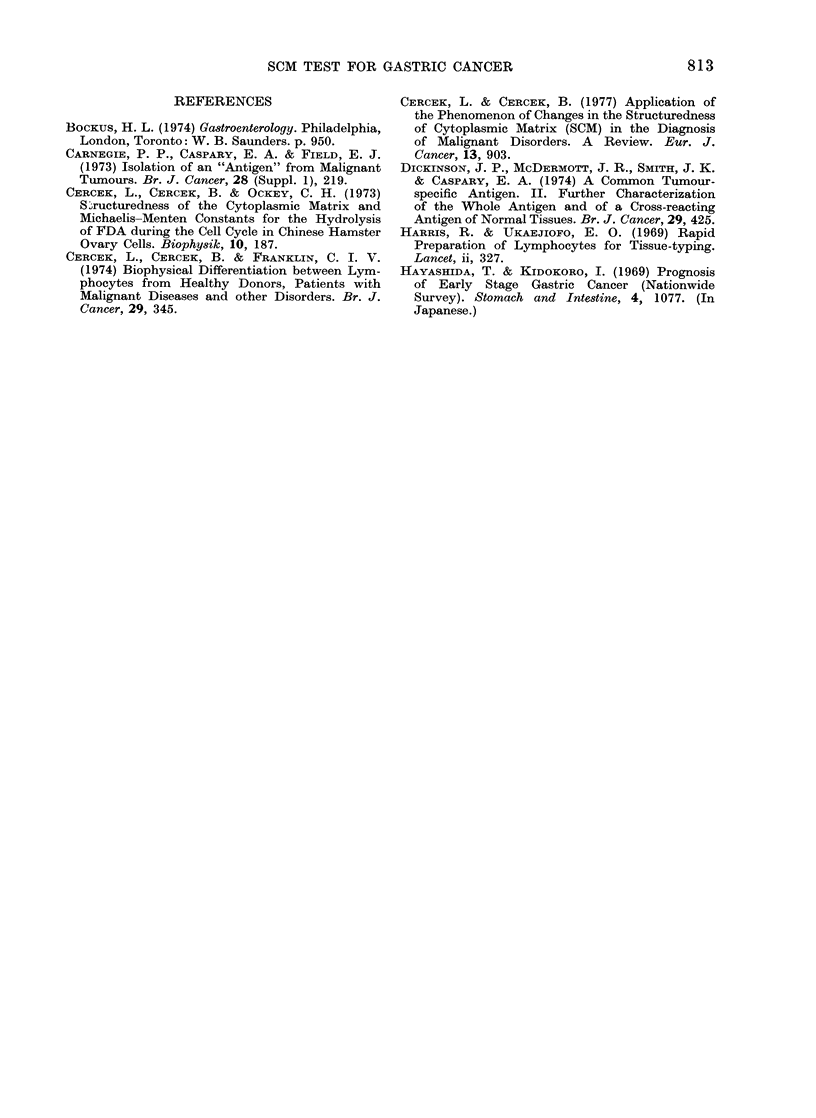# Usefulness of the SCM test in the diagnosis of gastric cancer.

**DOI:** 10.1038/bjc.1977.266

**Published:** 1977-12

**Authors:** F. Takaku, T. Yamanaka, Y. Hashimoto


Br. J. Cancer (1977) 36, 810

### Short Communication

### USEFULNESS OF THE SCM TEST IN THE DIAGNOSIS OF GASTRIC CANCER

F. TAKAKU*, T. YAMANAKA† AND Y. HASHIMOTO‡

*From the *Department of Medicine, Jichi Medical School, Tochigi, Japan, the †Department of Gastroenterology, Jichi Medical School, Tochigi, Japan, and the ‡3rd Department of Medicine, Faculty of Medicine, Tokyo University, Tokyo, Japan*

Received 26 May 1977 Accepted 2 August 1977

RECENTLY Cercek, Cercek and Franklin (1974) have shown that changes in the structuredness of cytoplasmic matrix (SCM) in human lymphocytes induced by cancer basic proteins (CaBP) and phytohaemagglutinin (PHA) can be used to differentiate patients with malignant disease from those with non-malignant disorders or healthy donors. Since the incidence of gastric cancer is very high in Japanese (Bockus, 1974) and the prognosis of gastrectomy in patients with the early stage of gastric cancer is reported to be excellent (Hayashida and Kidokoro, 1969) screening for the detection of the early stage of gastric cancer by regular X-ray examination of the stomach is conducted throughout Japan. To explore whether the SCM test can be used in the diagnosis of gastric cancer, we have performed 2 pilot studies, one involving 33 cases of cancer of the stomach, 15 healthy donors, 12 cases of non-malignant diseases and 39 cases of other malignant disorders, the other concentrating on 10 cases of "early stages" of cancer of the stomach. These were defined as "carcinoma of the stomach in which the invasion by cancer cells was limited to the mucosa and/or to submucosa", the definition being that of the Japanese Gastroenterological Endoscopy Society in 1962 and of the Japanese Research Society for Gastric Cancer in 1963. This definition is widely used in Japan.

All 10 patients were operated on and confirmed to be in the early stage of gastric cancer as defined above. Any cases found to have lymph-node metastasis on operation were excluded, even if the invasion by cancer cells was limited to the mucosa and/or submucosa, thus complying with the definition of early-stage gastric cancer.

Peripheral lymphocytes were isolated from heparinized blood (300 i.u. of heparin per 10 ml blood). Ten-millilitre aliquots of blood were rotated in glass vials containing 0·1 g of carbonyl iron powder at 60 rev/min for 30 min. Vials were then placed on a magnet for 10 min. Lymphocytes were separated by the Ficoll–Triosil gradient-density technique (Harris and Ukaejiofo, 1969). The density of the gradient was 1·081 at 25°C. The lymphocytes which band out on the interface were collected, washed twice with saline, once with Dulbecco's PBS, and resuspended in Dulbecco's PBS at the concentration of $6 \times 10^6$ cells/ml. Aliquots of 1 ml of lymphocyte suspensions were incubated at 37°C for 30 min with 50 μg of cancer basic protein (CaBP) and with 0·1 ml of a 50 × diluted reagent-grade PHA (Wellcome Ltd). The CaBP was extracted from colon cancer tissue according to published methods (Carnegie, Caspary and Field, 1973; Dickinson *et al.*, 1974). Aliquots of 0·2 ml of control or stimulated lymphocyte suspension were injected into 3 ml of 2·5 μM fluorescein-diacetate (FDA) solution in complete

Dulbecco's PBS (pH 7·4). The suspension was rapidly transferred into a 1 $cm^2$ cuvette and put into the thermostatted cuvette holder of the Hitachi MPF-4 fluorescence spectrophotometer fitted with the polarization accessory. Measurements were made at 27°C. Details of the SCM technique were the same as described by Cercek, Cercek and Ockey (1973) with the modifications of Cercek and Cercek (1977).

The sampling and measurements were performed by T.Y. and Y.H. The calculation of the results, however, was done by technicians who did not know the diagnosis of the patients. In some cases intermediate SCM values of around 1 were found, but on repeating the tests even these fell into a clearly positive or negative category. The reliability of the results appears to depend on the quality of the CaBP preparation. The first series of experiments was performed using a preparation obtained from a colon cancer. Results with new CaBP preparations from a case of gastric cancer gave smaller "stimulation".

The results of the first part of the study are shown in Tables I and II and the Figure, those of the second in Table III.

The mean value (*P*) of the SCM of unstimulated (control) lymphocytes from healthy donors was 0·236 ± 0·023, from patients with non-malignant disorders, 0·244 ± 0·026, and from patients with malignant disorders, 0·241 ± 0·028. Lymphocytes from healthy donors and patients with non-malignant diseases responded to PHA stimulation with decrease in the SCM to an average of 75% of control values. In contrast, lymphocytes from patients with malignant diseases either did not respond to PHA stimulation, or

TABLE I.—*SCM Response of Lymphocytes from Patients with Stomach Cancer*

| Case No. | Age | Sex | Diagnosis | $RR_{SCM}$ |
|---|---|---|---|---|
| 1 | 35 | M | Ca (early) | 0·86 |
| 2 | 43 | F | Ca (early) | 0·83 |
| 3 | 48 | M | Ca (early) | 0·79 |
| 4 | 57 | F | Ca (early) | 0·86 |
| 5 | 68 | M | Ca (early) | 0·91 |
| 6 | 73 | M | Ca (advanced) | 0·79 |
| 7 | 64 | M | Ca (advanced) | 0·66 |
| 8 | 58 | M | Ca (advanced) | 0·67 |
| 9 | 73 | M | Ca (advanced) | 0·86 |
| 10 | 46 | M | Ca (advanced) | 0·68 |
| 11 | 57 | M | Ca (advanced) | 0·70 |
| 12 | 65 | M | Ca (advanced) | 0·85 |
| 13 | 75 | M | Ca (advanced) | 0·69 |
| 14 | 41 | F | Ca (advanced) | 0·90 |
| 15 | 45 | M | Ca (advanced) | 0·71 |
| 16 | 49 | M | Ca (advanced) | 0·85 |
| 17 | 58 | M | Ca (advanced) | 0·88 |
| 18 | 59 | F | Ca (advanced) | 0·85 |
| 19 | 63 | F | Ca (advanced) | 0·84 |
| 20 | 55 | M | Ca (advanced) | 0·80 |
| 21 | 45 | M | Ca (advanced) | 0·75 |
| 22 | 52 | M | Ca (advanced) | 0·72 |
| 23 | 58 | F | Ca (advanced) | 0·79 |
| 24 | 70 | M | Ca (advanced) | 0·93 |
| 25 | 48 | F | Ca (early) | 0·69 |
| 26 | 71 | F | Ca (advanced) | 0·89 |
| 27 | 60 | M | Ca (advanced) | 0·89 |
| 28 | 35 | F | Ca (advanced) | 0·71 |
| 29 | 38 | M | Ca (advanced) | 0·77 |
| 30 | 60 | M | Ca (advanced) | 0·87 |
| 31 | 35 | M | Leiomyosarcoma (advanced) | 0·86 |
| 32 | 41 | M | Adenocarcinoma (advanced) | 0·99 |
| 33 | 41 | M | Adenocarcinoma (advanced) | 0·76 |

TABLE II.—*SCM Response of Lymphocytes from Patients with Non-malignant Diseases and Healthy Donors*

| Case No. | Age | Sex | Diagnosis | $RR_{SCM}$ |
|---|---|---|---|---|
| 1 | 26 | M | Healthy | 1·50 |
| 2 | 28 | M | Healthy | 1·32 |
| 3 | 28 | M | Healthy | 1·31 |
| 4 | 37 | F | Healthy | 1·55 |
| 5 | 64 | M | Healthy | 1·49 |
| 6 | 27 | M | Healthy | 1·50 |
| 7 | 28 | M | Healthy | 1·40 |
| 8 | 28 | M | Healthy | 1·40 |
| 9 | 29 | M | Healthy | 1·20 |
| 10 | 20 | M | Healthy | 1·21 |
| 11 | 34 | F | Healthy | 1·32 |
| 12 | 34 | F | Healthy | 1·08 |
| 13 | 35 | M | Healthy | 1·29 |
| 14 | 54 | M | Healthy | 1·10 |
| 15 | 60 | F | Healthy | 1·10 |
| 16 | 6 | M | Schilder's disease | 1·23 |
| 17 | 9 | M | Schilder's disease | 1·55 |
| 18 | 40 | F | Acute hepatitis | 1·55 |
| 19 | 63 | M | Acute hepatitis | 1·32 |
| 20 | 49 | F | Cholelithiasis | 1·59 |
| 21 | 26 | M | Pregnancy (34 weeks) | 1·34 |
| 22 | 25 | F | Pregnancy (28 weeks) | 1·41 |
| 23 | 70 | M | Lymphadenitis | 1·31 |
| 24 | 28 | F | Systemic lupus erythematosus | 1·06 |
| 25 | 23 | F | Systemic lupus erythematosus | 1·29 |
| 26 | 52 | M | Rheumatoid arthritis | 1·24 |
| 27 | 32 | F | Iron deficiency anaemia | 1·08 |

FIG. Distribution of $RR_{SCM}(P_{CaBP}/P_{PHA})$ for 39 cases of malignancy other than stomach cancer.

showed a < 5%, decrease in the SCM value. Lymphocytes from healthy donors and from patients with non-malignant disorders did not respond to CaBP, even after up to 180 min of incubation. In contrast, lymphocytes from patients with malignant disorders responded to CaBP with an average decrease in the SCM of 20%. Changes in the SCM response of lymphocytes to CaBP and PHA are presented as the SCM response ratio: $RR_{SCM} = P_{CaBP}/P_{PHA}$ (Cercek *et al.*, 1974). The $RR_{SCM}$ values in lymphocytes from healthy donors and patients with non-malignant diseases range from 1·06 to 1·55 and for all patients with cancer from 0·66 to 0·99. Details of 33 patients with gastric cancer are presented in Table I. The results obtained with 39 cases of other malignant disorders (*viz.* cancers of the oesophagus, duodenum, colon, gall bladder, common bile duct, pancreas, larynx, lung and breast) are summarized as an $RR_{SCM}$ histogram in the Figure. Details for healthy donors and patients with non-malignant disorders are described in Table II, for the 10 cases of early cancer in Table III.

These pilot investigations indicate the usefulness of the SCM test for early diagnosis of cancer, and we are now collecting further cases for the quantitative evaluation of the accuracy of the test.

We are obliged to Drs L. and B. Cercek of the Paterson Laboratories, Christie Hospital and Holt Radium Institute, Manchester, England for technical information and advice on the SCM technique.

We thank the surgeons of the Second Department of Surgery, Faculty of Medicine, University of Tokyo for their collaboration.

TABLE III.—*$RR_{SCM}$ of Lymphocytes and Histological Findings of Early-Stage Cancer of the Stomach*

| Case No. | Age | Sex | Histological diagnosis | Size of tumour (cm) | Invasion limited to | Permeation to: Lymph vessel | Permeation to: Vein | Lymph node metastasis | $RR_{SCM}$ |
|---|---|---|---|---|---|---|---|---|---|
| 1 | 35 | M | Adenoca. mucocellulare et microtubulare | 2·5×1·0 | Mucosa | — | — | — | 0·86 |
| 2 | 43 | F | Adenoca. mucocellulare | 3·5×1·5 | Submucosa | + | — | — | 0·83 |
| 3 | 48 | M | Adenoca. mucocellulare | 1·8×1·6 | Mucosa | — | — | — | 0·82 |
| 4 | 57 | M | Adenoca. tubulare | 0·8×1·4 | Mucosa | — | — | — | 0·86 |
| 5 | 67 | M | Adenoca. tubulare | (2·0×0·8, 0·5×0·5)† | Mucosa | — | — | — | 0·91 |
| 6 | 28 | M | Adenoca. mucocellulare | 2·5×3·5 | Mucosa | — | — | — | 0·83 |
| 7 | 42 | F | Adenoca. mucocellulare | 10·5×5·5* | Mucosa | — | — | — | 0·91 |
| 8 | 81 | F | Adenoca. tubulare | 1·2×2·5 | Mucosa | — | — | — | 0·79 |
| 9 | 50 | F | Adenoca. tubulare | 6·0×7·0† | Mucosa | — | — | — | 0·81 |
| 10 | 68 | F | Adenoca. tubulare | 2·5×2·0 | Mucosa | — | — | — | 0·73 |
| Normal and non-malignant (26 cases) | | | | | | | | | 1·33 ±0·14 |

* Double cancer.

† Superficial infiltration confined to mucosa.

### REFERENCES

Bockus, H. L. (1974) *Gastroenterology*. Philadelphia, London, Toronto: W. B. Saunders. p. 950.

Carnegie, P. P., Caspary, E. A. & Field, E. J. (1973) Isolation of an "Antigen" from Malignant Tumours. *Br. J. Cancer*, **28** (Suppl. 1), 219.

Cercek, L., Cercek, B. & Ockey, C. H. (1973) Structuredness of the Cytoplasmic Matrix and Michaelis–Menten Constants for the Hydrolysis of FDA during the Cell Cycle in Chinese Hamster Ovary Cells. *Biophysik*, **10**, 187.

Cercek, L., Cercek, B. & Franklin, C. I. V. (1974) Biophysical Differentiation between Lymphocytes from Healthy Donors, Patients with Malignant Diseases and other Disorders. *Br. J. Cancer*, **29**, 345.

Cercek, L. & Cercek, B. (1977) Application of the Phenomenon of Changes in the Structuredness of Cytoplasmic Matrix (SCM) in the Diagnosis of Malignant Disorders. A Review. *Eur. J. Cancer*, **13**, 903.

Dickinson, J. P., McDermott, J. R., Smith, J. K. & Caspary, E. A. (1974) A Common Tumour-specific Antigen. II. Further Characterization of the Whole Antigen and of a Cross-reacting Antigen of Normal Tissues. *Br. J. Cancer*, **29**, 425.

Harris, R. & Ukaejiofo, E. O. (1969) Rapid Preparation of Lymphocytes for Tissue-typing. *Lancet*, ii, 327.

Hayashida, T. & Kidokoro, I. (1969) Prognosis of Early Stage Gastric Cancer (Nationwide Survey). *Stomach and Intestine*, **4**, 1077. (In Japanese.)